# miRNA-338-3p inhibits the migration, invasion and proliferation of human lung adenocarcinoma cells by targeting MAP3K2

**DOI:** 10.18632/aging.204198

**Published:** 2022-08-03

**Authors:** Bo Zhang, Dongchang Wang, Yuanyuan Wang, Gang Chen

**Affiliations:** 1 Department of Geriatrics, The Third Hospital of Hebei Medical University, Shijiazhuang, China; 2 Department of Respiratory Medicine, The Third Hospital of Hebei Medical University, Shijiazhuang, China

**Keywords:** miR-338-3p, lung adenocarcinoma cell, MAP3K2, migration, invasion

## Abstract

Objective: This study aimed to investigate the effects of micro ribonucleic acid (miR)-338-3p on the migration, invasion and proliferation of lung adenocarcinoma (LUAD) cells.

Methods: Bioinformatics analysis was employed to evaluate the function and expression of related genes in lung cancer. Human A549 and NCI-H1299 cells cultured to logarithmic growth stage were assigned to negative control (NC) mimic group, miR-338-3p mimic group (miR-mimic group), NC inhibitor group and miR-338-3p inhibitor group (miR-inhibitor group) treated with or without MAP3K2 overexpression (OE)-lentivirus, or TBHQ or FR180204. Transwell assay, cell colony formation assay, Western blotting and cell-cycle analysis were carried out.

Results: Bioinformatics results manifested that miR-338 and MAP3K2 were involved in LUAD. The expression levels of MAP3K2, p-ERK1/2, MMP-2, MMP-3, MMP-9, cyclin A2 and cyclin D1 were increased after addition of miR-338-3p inhibitor, consistent with the raised amount of LUAD cells in migration and invasion experiments and number of colonies formed, as well as the cell cycle, but miR-338-3p mimic reversed these results. Moreover, MAP3K2 overexpression elevated the level of p-ERK1/2. Meanwhile, after treatment with TBHQ or FR180204, the influence of miR-338-3p inhibitor or mimic was also verified.

Conclusions: MiR-338-3p overexpression can modulate the ERK1/2 signaling pathway by targeting MAP3K2, thus inhibiting the migration, invasion and proliferation of human LUAD cells.

## INTRODUCTION

Lung cancer is one of the primary causes of death in China. Lung adenocarcinoma (LUAD) ranks first among all subtypes in respect of incidence rate and the number of LUAD cases shows an uptrend in recent years [1, 2], posing serious threats to the health of patients. Invasion and metastasis of LUAD are main factors resulting in death [3], and most of such patients suffer from advanced non-small cell lung cancer (NSCLC) [4]. Histologically, NSCLC can be categorized into LUAD and squamous-cell carcinoma, the former of which frequently occurs and accounts for more than 50% of all subtypes [5]. Considering that LUAD exhibits an annually increasing prevalence, it is necessary to explore the molecular mechanism of invasion and metastasis of LUAD cells, so as to improve the quality of life of LUAD patients.

Micro ribonucleic acids (miRNAs) negatively regulate gene expression through complementarity to the 3′-untranslated region (3′-UTR) of target gene mRNAs [6]. MiRNAs are implicated in multiple cellular and physiological processes such as cell epithelial-mesenchymal transition, and their abnormal expressions are related to the progression and metastasis of various human tumors [7]. MiR-338-3p is lowly expressed in several tumors, and up-regulating miR-338-3p can inhibit the expression of certain target genes, which plays an important role in anti-tumor cell proliferation and may become a novel target for clinical treatment of tumors. Specifically, miR-338-3p is capable of suppressing the growth and inducing the apoptosis of intestinal cancer cells through inhibiting the phosphorylated extracellular signal-regulated kinase ½ (p-ERK1/2) and P38 proteins in the mitogen-activated protein kinase (MAPK) pathway [8], similar to findings in colorectal cancer and cervical cancer. Furthermore, miR-338-3p is also able to negatively regulate MACC1 and then inhibit the level of p-ERK1/2 in the MAPK pathway to induce tumor cell apoptosis and inhibit proliferation [9, 10]. However, the specific role of miR-338-3p in LUAD remains elusive.

Therefore, the expression of miR-338-3p in LUAD was analyzed through the GEO database and TCGA database, and bioinformatics technology was utilized for pathway enrichment analysis. Besides, the specific regulatory mechanism of miR-338-3p/mitogen-activated protein kinase kinase kinase 2 (MAP3K2) on the migration, invasion and proliferation of LUAD cells was investigated through basic cell experiments, so as to provide potentially valuable biomarkers for the clinical evaluation of migration, invasion and proliferation of LUAD cells.

## METHODS

### Bioinformatics analysis

Firstly, the LUAD-related gene expression dataset GSE136043 and the miRNA sequencing dataset GSE24709 containing data of LUAD patients were downloaded from Gene Expression Omnibus (GEO) database (https://www.ncbi.nlm.nih.gov/gds/). Then the quantile normalization was performed on RNA-seq data and differentially expressed genes (DEGs) (|logFC|>1, *P* < 0.05) using limma (R/Bioconductor software package). Later, a volcano plot was constructed to visualize the DEGs in GSE136043 using R with ggplot2 package, and a cluster analysis heatmap of DEGs was plotted in R with pheatmap package. In the same way, DEGs in GSE24709 were visualized by the volcano plot, and the cluster analysis was conducted using the heatmap.

### Functional enrichment analysis

Gene Ontology (GO) and Kyoto Encyclopedia of Genes and Genomes (KEGG) enrichment analyses were performed for DEGs obtained from GSE136043. The DEGs corresponding to biological processes, cell components and molecular functions were analyzed by the online database tool DAVID (https://david.ncifcrf.gov/) to integrate GO terms and create a biological process network of DEGs. After that, a GO pathway diagram and a KEGG pathway enrichment analysis diagram of DEGs were drawn in R programming with GOplot and ggplot2 packages. Through protein-protein interaction (PPI) database (https://www.string-db.org/) and the string database, the differential genes were subjected to generate the interaction network. DEGs were input into string online tool to screen interaction proteins. The PPI network was established and visualized by the software in the PPI network constructed by Cytoscape, and the degree of correlation between different genes was determined by using the molecular complex detection (MCODE) plug-in. In the most significant module, the cytoHubba plug-in was applied to calculate the hub genes with the top scores according to the degree algorithm.

### Gene set enrichment analysis (GSEA)

GSEA (http://www.gsea-msigdb.org/) was carried out for all genes, and the corresponding pathway diagram was plotted.

### miRNA target gene prediction

The target genes of miRNA were predicted using miRNA target databases miRDB and TargetScan, and the interaction pairs of differentially expressed miRNA and mRNA were obtained in combination with GSE136043. Moreover, miRNA-mRNA binding sites were mapped using the Wayne tool VennDiagram package.

### Cell culture and grouping

In this study, human LUAD A549 and NCI-H1299 [11] cells were cultured in RPMI 1640 medium containing 10% fetal bovine serum (Beijing Solarbio Science and Technology Co., Ltd.) in an incubator with 5% CO_2_ at 37°C for 24 hours. Firstly, the cells were transfected according to the instructions of Lipofectamine™ 2000 transfection reagent. Then, the cells cultured to the logarithmic growth phase were taken, digested with 2.5% trypsin, collected, and inoculated in a six-well plate. When cell confluence reached 50%, cell transfection was performed, and the corresponding plasmids were transferred into A549 cells to divide them into four groups: NC mimic group, miR-mimic group, NC inhibitor group and miR-inhibitor group, with NC mimic group and NC inhibitor group as negative controls for miR-mimic group and miR-inhibitor group.

### Transwell migration and invasion experiments

The migration and invasion abilities of A549 cells were analyzed by Transwell experiments. In this case, the cells were resuspended in serum-free medium. The cells at a concentration of 5 × 10^5^ cells/mL were added to the Transwell upper chamber, and the complete medium containing 10% fetal bovine serum was added to the lower chamber. Unlike the migration experiment, the upper chamber was coated with Matrigel in the Transwell invasion experiment, and the remaining steps were identical to those in the cell migration experiment. After 24 hours of incubation at 37°C, the Transwell chamber was removed for routine fixation, and stained with crystal violet, after which the excess crystal violet was discarded. After drying, the cells on the bottom membrane of the chamber were observed and counted under a microscope.

### Dual luciferase assay

According to the prediction based on the TargetScan database (release 7.2; http://www.targetscan.org/), a putative miR-338-3p-binding site (UGCUGGA) was identified in the 3′-UTR of the MAP3K2 gene1.9. Wild-type (WT) and mutant (MUT) 3′-UTR sequences were amplified and cloned by polymerase chain reaction (PCR). PGL3 dual-luciferase report vectors pGL3-MAP3K2-3′UTR-WT and pGL3-MAP3K2-3′UTR-MUT were created by Hebei Xunyue Biology Co., Ltd. Subsequently, the constructed plasmid was mixed with miR-338-3p (NC/mimic) and co-transfected into NCI-H1299 and A549 cells for 48 hours. Later, the cells in each group were digested with 0.25% EDTA trypsin, collected and added with 500 μL of lysate, followed by incubation on ice for 5 min. After that, the cells were fully lysed, and 20 μL of cell lysate was added to the black microplate and mixed with 100 μL of firefly luciferase reaction solution by shaking, so as to detect the activity of firefly luciferase. Meanwhile, the microplate was mixed with 100 μL of Renilla luciferase reaction solution by shaking to detect the activity of Renilla luciferase. Three replicates were set for each experiment in each group.

### Quantitative PCR (qPCR)

Cells in each group were digested with 0.25% EDTA trypsin and collected, and TRIzol was added to extract total RNA, whose purity and concentration were detected by an UV spectrophotometer. Later, the total RNA was reversely transcribed into cDNA on a 20 μL reverse transcription system: 4 μL of 5× RT buffer, 0.5 μL of RNase inhibitors, 0.5 μL of Oligo (dT), 0.5 μL of ReverTra Ace and DEPC-treated water to a final volume of 20 μL. QPCR was carried out in a fluorescence qPCR instrument, which involved an initial denaturation at 98°C for 2 min, followed by 40 cycles of denaturation at 98°C for 10 s, annealing at 55°C for 15 s, and extension at 72°C for 30 s. The obtained cDNA was subjected to qPCR in the following system (10 μL): 1 μL of cDNA, 0.5 μL of upstream primers, 0.5 μL of downstream primers, 5 μL of 2× TransStart SYBR Green qPCR Supermix, and 3 μL of ddH_2_O. Each experiment was carried out 4 times, with 3 replicates for each sample. Primer sequences of hsa-miR-338-3p and GAPDH used in this study are listed as follows:

**Table:** 

hsa-miR-338-3p	F: 5′-ATATCCTGGTGCTGAGTG-3′
	R: 5′-GAACATGTCTGCGTATCTC-3′
GAPDH	F: 5′-GGCACCCAGCACAATGAAG-3′
	R: 5′-CCGATCCACACGGAGTACTTG-3′

### Cell colony formation assay

Cell colony formation assay was performed. In detail, after 48 hours of transfection, the cells were collected and digested with trypsin. Then they were seeded in a six-well plate at a concentration of 2 × 10^2^ cells/well, with 3 parallel wells in each group, and cultured in a 37°C incubator for 1 week. After that, the cells were transfected again, and the clonal cell clusters could be observed with naked eyes at about 2 weeks after culture. Next, the culture medium was discarded, and the cells were washed 3 times with PBS, fixed with methanol for 15 min, dried and stained with hematoxylin for 30 min. After drying, scanning and photographing were carried out, and the clonal clusters visible to the naked eye were counted.

### Western blotting assay

Firstly, the whole-cell protein extract was lysed in a six-well plate with lysis buffer, homogenized, and centrifuged at 12,000 rpm for 15 min at 4°C, so as to determine the protein concentration by the BCA method. After electrophoresis and subsequent transfer, the cells were incubated with primary antibodies against MAP3K2, p-ERK1/2, total ERK1/2 (t-ERK1/2), matrix metalloproteinase (MMP)-2, MMP-3, MMP-9, cyclin A2, cyclin D1, and β-actin (1:1,000) overnight at 4°C, and incubated with secondary antibodies (1:5,000) for 2 hours at room temperature. Finally, the protein bands were visualized with a high-efficiency chemiluminescence solution, with β-actin as an internal control.

### Cell cycle analyzed by flow cytometry

The cells were inoculated into 6 well plates, cultured to a concentration of 30% ~ 50%, and then transfected with NC, mimic and inhibitor for 48 hours, the cells were collected, washed with PBS buffer and fixed in 70% ethanol at 4°C overnight. RNA was removed with RNase A at 37°C for 30 min. Wash off the fixative and add 300 μL PI/RNase A staining working solution, incubate at room temperature in the dark for 30 ~ 60 min. Check on the machine. The cell cycle distribution was analyzed by ModFit LT software. The experiment was repeated three times.

### Human specimens

Part of human lung adenocarcinoma and its pericancerous tissues (*n* = 14) were pumped with formaldehyde and then stained with HE, and the other were stored in liquid nitrogen and tested by Q-PCR to evaluate the levels of miR--338-3p, all the all the experimental operations for human specimens were under the ethical requirements of the Second Affiliated Hospital of Hebei Medical University.

### Statistical analysis

Statistical analysis of the bioinformatics results was conducted using R v3.6.1 with DEseq2 and ggpubr packages. Wald test was adopted for differential gene analysis, and rank sum test for comparison of cytokines between two groups. All experimental data were expressed by mean ± standard deviation (Mean ± SD) and analyzed by independent-samples *t*-test. *P* < 0.05 indicated that the difference was statistically significant.

## RESULTS

### Screening of DEGs

**Figure 1:**
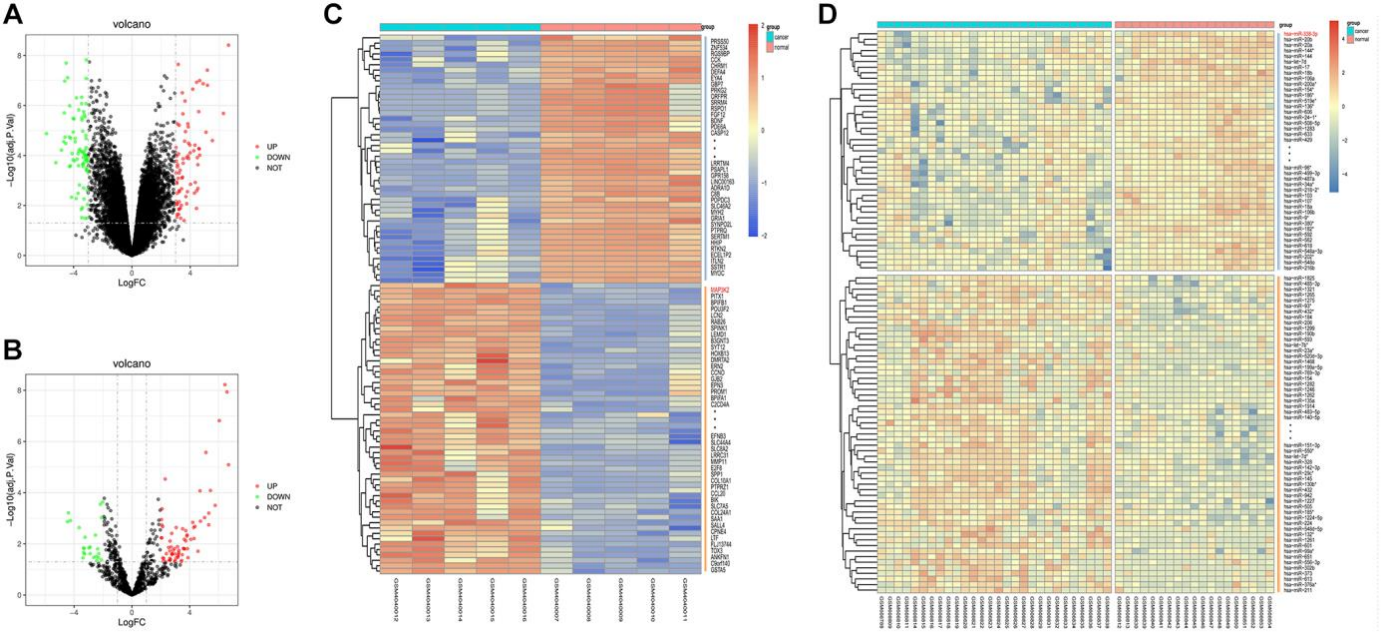
**Screening of differentially expressed genes (DEGs).** (**A**, **B**) Volcano plots of visually grouped DEGs in GSE136043 and GSE24709. (**C**, **D**) Cluster analysis heatmaps of GSE136043 and GSE24709.

The screening of LUAD-related GSE136043 downloaded from the GEO database was completed according to the criteria of *P* < 0.05 and |logFC|>1. The results showed that there were 153 DEGs in LUAD mRNAs, out of which 75 DEGs were up-regulated and 78 DEGs were down-regulated. Then, a volcano plot (Figure 1A) was drawn to visualize DEGs in GSE136043 using R with ggplot2 package, and a cluster analysis heatmap of DEGs was plotted in R with pheatmap package (Figure 1B), illustrating that MAP3K2 is highly expressed in cancer group. In the same way, quantile normalization of GSE24709 and screening of DEGs were performed, and the volcano plot (Figure 1C) and cluster analysis heatmap (Figure 1D) of DEGs were drawn.

### Bioinformatics analysis

**Figure 2:**
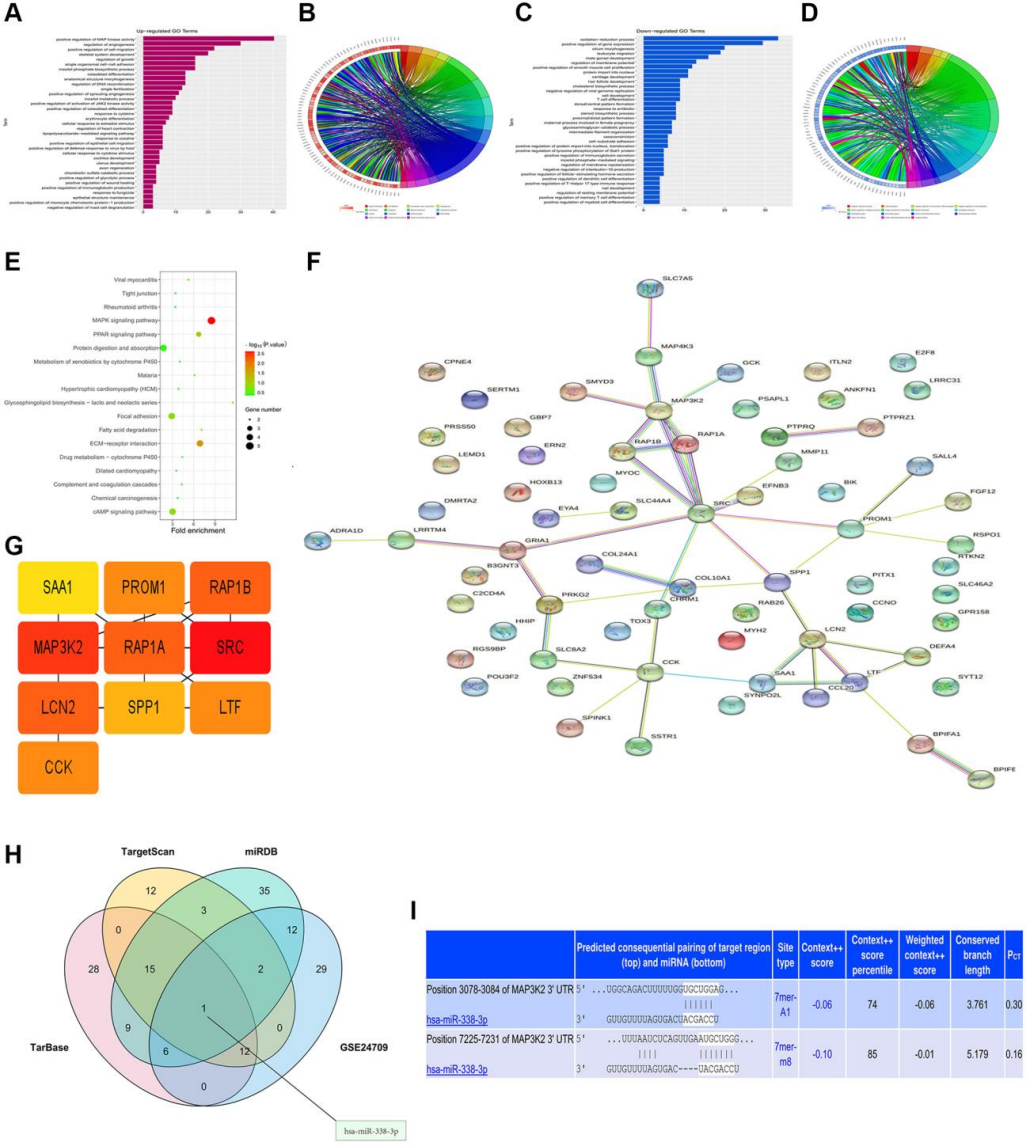
**Bioinformatics analysis and prediction of miRNA target genes.** (**A**, **B**) Up-regulated GO pathway of DEGs. (**C**, **D**) Down-regulated GO pathway of DEGs. (**E**) KEGG pathway chart. (**F**, **G**) Venn diagram and miRNA-mRNA binding sites (**H**, **I**).

The diagrams of up-regulated (Figure 2A, 2B) and down-regulated (Figure 2C, 2D) GO pathways of DEGs were plotted by R programming. The GO pathway diagrams illustrated that both up-regulated pathways (positive regulation of MAPK activity, regulation of angiogenesis, and positive regulation of cell migration) and down-regulated pathways (oxidation-subtraction process, positive regulation of gene expression, and silicon morphogenesis) were enrichment pathways for LUAD. Moreover, the KEGG pathway diagram of DEGs (Figure 2E) revealed that the MAPK signaling pathway, ECM-sensor interaction and other signaling pathways were enriched. PPI analysis for DEGs in GO pathway was carried out by string online tool and Cytoscape software (Figure 2F). In the PPI network established based on string database, the most significant modules were selected using the MCODE plug-in in Cytoscape. For the most significant module, the cytoHubba plug-in was used to calculate the top 10 key genes with the degree algorithm, including MAP3K2 (Figure 2G).

### miRNA target gene prediction

The miRNA target gene prediction is a necessary link to investigate the functional mechanism of miRNAs. Accurate target gene prediction can save the time and costs of later experimental verification and improve the verification efficiency. The candidate target genes of miRNAs and the binding sites of miRNAs in mRNAs were predicted by TargetScan, miRDB and TarBase online databases. The results manifested the binding sites of miRNAs in mRNAs among different species and the thermal stability of double-strand RNAs. Besides, it was also found that there was no complex secondary structure at the binding sites of miRNAs. Subsequently, based on TargetScan, miRDB and TarBase databases, mRNAs and miRNAs were predicted, respectively. Then VennDiagram package was utilized to draw a Venn diagram of DEGs in GSE24709, and the intersection was taken to determine the co-binding miRNA, namely miR-338-3p (Figure 2H). The target gene 3′-UTR and miRNAs were predicted by searching the conserved 7mer-A1 and 7mer-m8 sites matching each miRNA seed region in the TargetScan online database, and the binding site of MAP3K2 to miR-338-3p was determined (Figure 2I).

### GSEA

**Figure 3:**
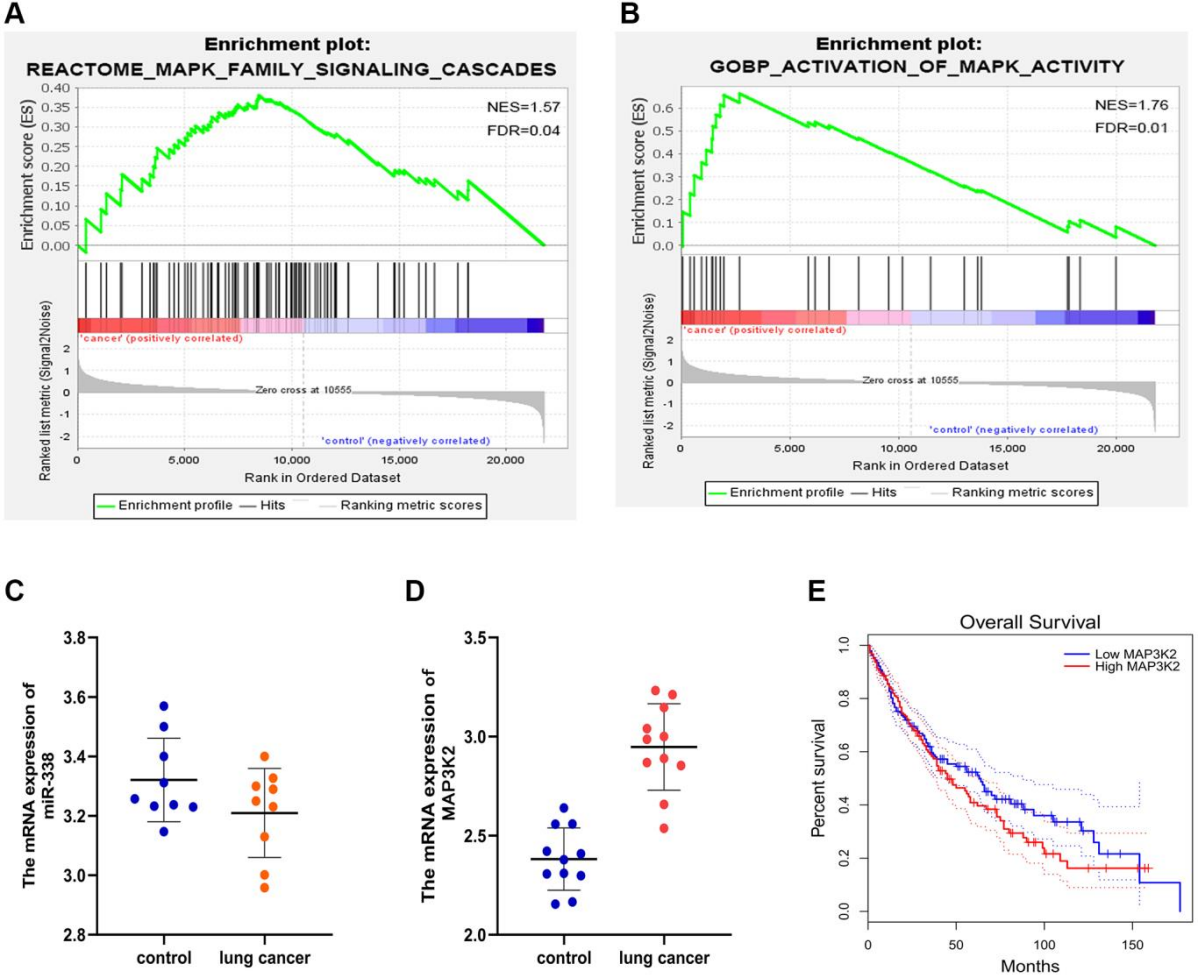
**GSEA and target gene statistical analysis.** (**A**, **B**) GSEA. (**C**, **D**) Expressions of miR-338-3p and MAP3K2 in lung cancer. (**E**) Survival curves.

GSEA results demonstrated the enrichment in the MAPK family signal cascades and the activated MAPK activity pathway (Figure 3A, 3B).

### Statistical analysis of target genes

The analysis of LUAD-related genes from GEO and TCGA databases showed that miR-338-3p was lowly expressed (Figure 3C), and MAP3K2 was highly expressed (Figure 3D) in LUAD tissues. Besides, it was also discovered that the highly-expressed MAP3K2 displayed a relevance to a low survival rate (Figure 3E).

### The expression of miR-338-3p in human lung adenocarcinoma and relationship between miR-338-3p and MAP3K2

**Figure 4:**
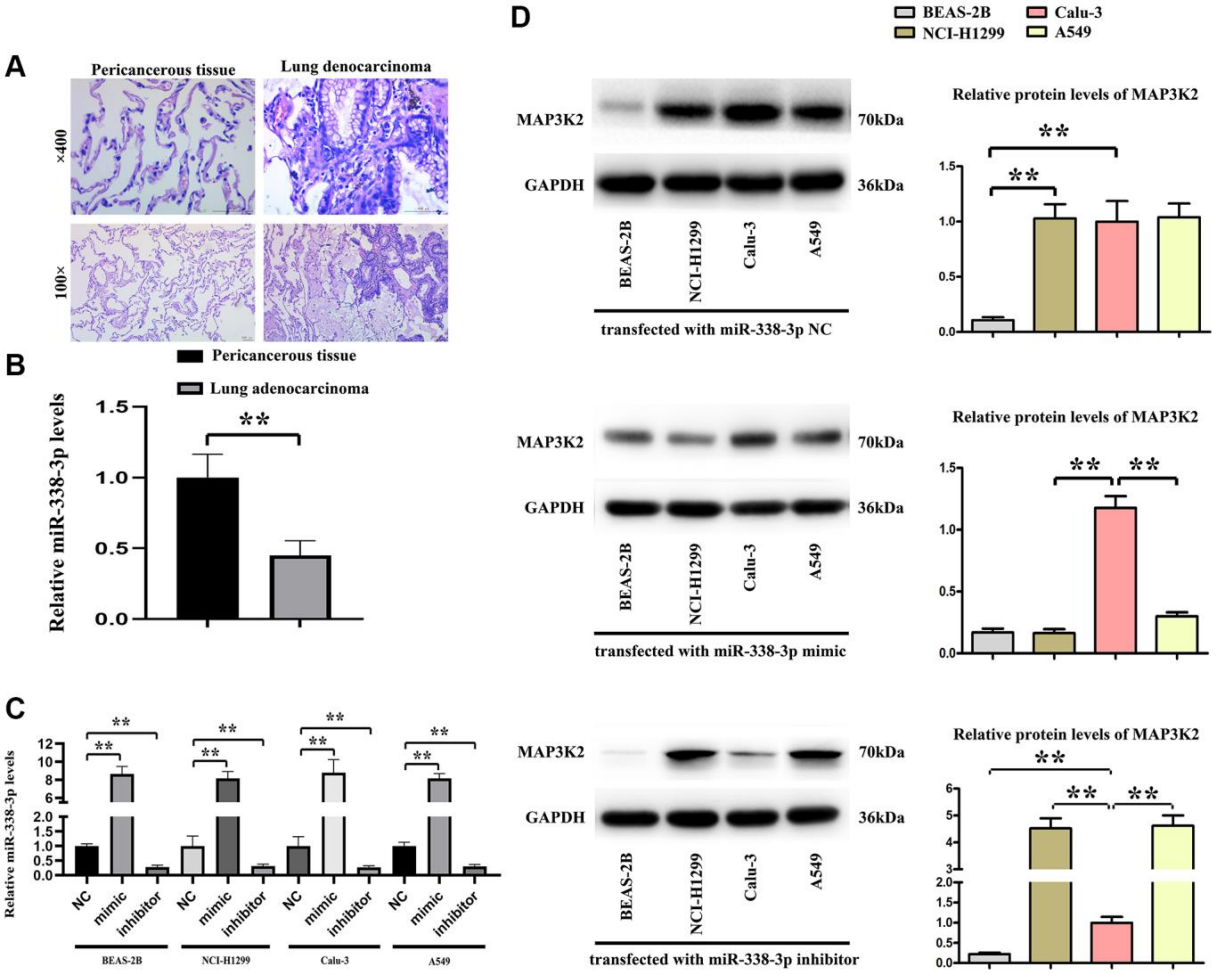
**The relationship of miR-338-3p with MAP3K2 in human specimens.** (**A**) Human lung adenocarcinoma and pericancerous tissue were identified by HE staining. (**B**) The statistical decreased expression of miR-338-3p in adenocarcinoma than pericancerous. ^**^P < 0.01 means that the difference is statistically significant. (**C**) The relative miR-338-3p levels in 4 cell lines after transfected with miR-338-3p NC, mimic, inhibitor. ^**^P < 0.01 means that the difference is statistically significant. (**D**) miR-338-3p mimic decreased the MAP3K2 protein levels, and miR-338-3p inhibitor increased the MAP3K2 protein levels in human adenocarcinoma cell lines: NCI-H1299 and A549 cells but not Calu-3 cells. ^**^P < 0.01 vs. Calu-3 group, means that the difference is statistically significant.

The human specimens of lung adenocarcinoma and its pericancerous tissue were identified by HE staining and shown in Figure 4A, the relative expression levels of miR-338-3p were tested by Q-PCR and our results showed the significantly decreased miR-338-3p were found in lung adenocarcinoma tissue vs pericancerous tissue, shown in Figure 4B, and then the normal human BEAS-2B cells are epithelial cells, isolated from pathological sections of normal human bronchial epithelium, and human adenocarcinoma cell lines, included the NCI-H299, Calu-3, A549 cells were transfected with miR-338-3p mimic and inhibitor. The relative miR-338-3p levels were tested by Q-PCR and results were showed in Figure 4C, indicating the transfection efficiency was effective. Then the protein levels of MAP3K2 were tested by western blot, the results showed the MAP3K2 proteins were negatively related the expression of miR-338-3p in NCI-H299, A549 cells but not in BEAS-2B and Calu-3 cells, and the results were shown in Figure 4D.

### Expression of miR-338-3p/MAP3K2 axis in NCI-H1299 and A549 cells

**Figure 5:**
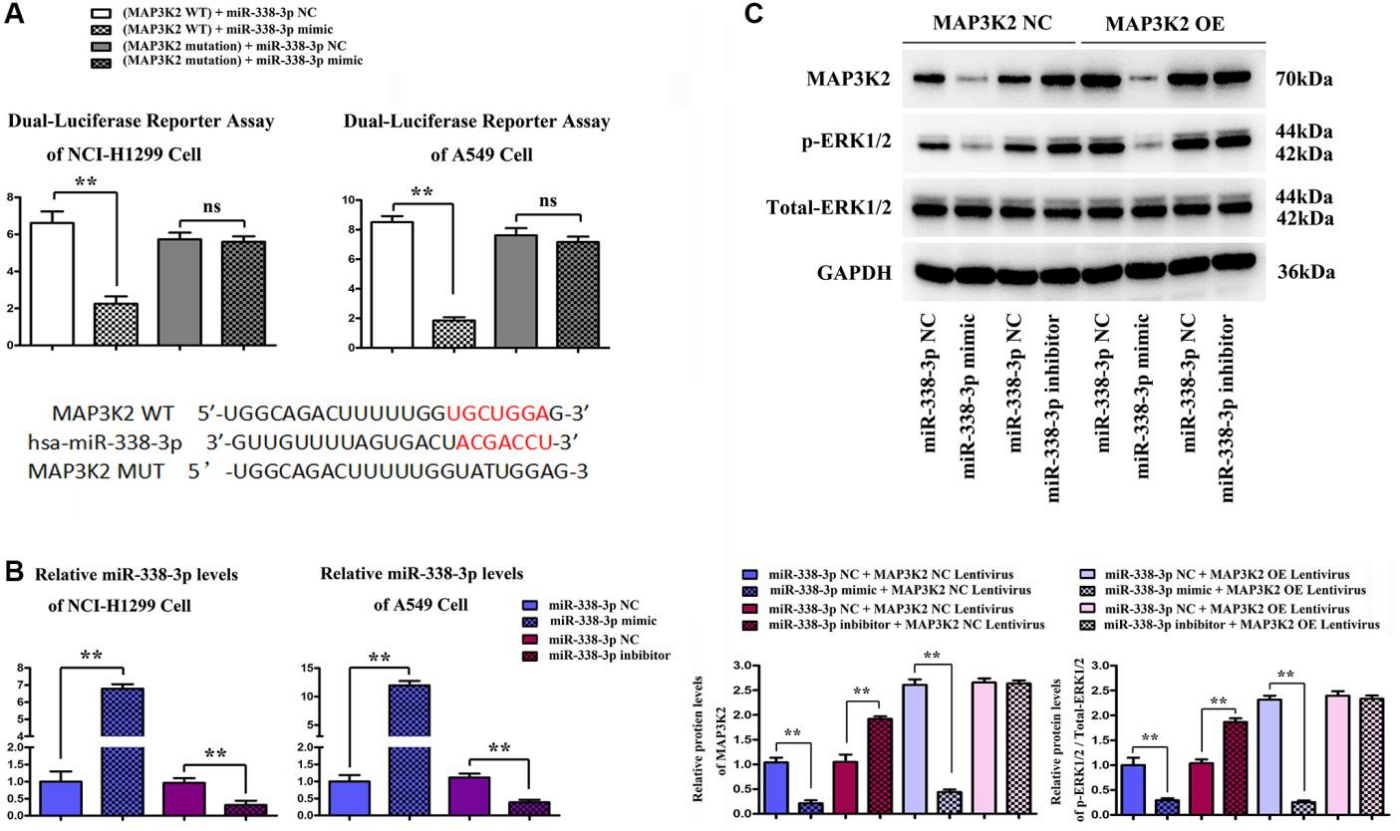
**Functional verification of miR-338-3p/MAP3K2 axis in NCI-H1299 and A549 cells.** (**A**) the effect of miR-338-3p on MAP3K2 transcription was identified by luciferase reporter assay in HCI-H1299 and A549 cells, and the putative wild-type MAP3K2 3'-UTR binding sequence as well as the mutation sequence. (**B**) Statistical results of protein expressions in NCI-H1299 and A549 cells. ^**^P < 0.01 means that the difference is statistically significant. (**C**) Protein expressions of MAP3K2, p-ERK1/2 and t-ERK1/2 in A549 cells. ^**^P < 0.01 means that the difference is statistically significant.

The effect of miR-338-3p on MAP3K2 transcription was investigated by luciferase reporter assay in HCI-H1299 and A549 cells, as well as the putative wild-type MAP3K2 3′-UTR binding sequence as well as the mutation sequence were shown in Figure 5A. The miR-338-3p mimic, inhibitor and negative controls (NC) were transfected into HCI-H1299 and A549 cells and selective miR-338-3p expression levels are shown in Figure 5B. After transfection of A549 cells with miR-inhibitor, MAP3K2 protein level was significantly increased, while p-ERK1/2 was also increased. However, after exogenous overexpression of miR-338-3p, the protein expressions of MAP3K2 and p-ERK1/2 were significantly reversed. Moreover, it was found that after under miR-338-3p inhibited state, the protein expressions of MAP3K2 and p-ERK1/2 among groups were also elevated (Figure 5C).

### Overexpression of miR-338-3p inhibited the migration and invasion of LUAD cells

**Figure 6:**
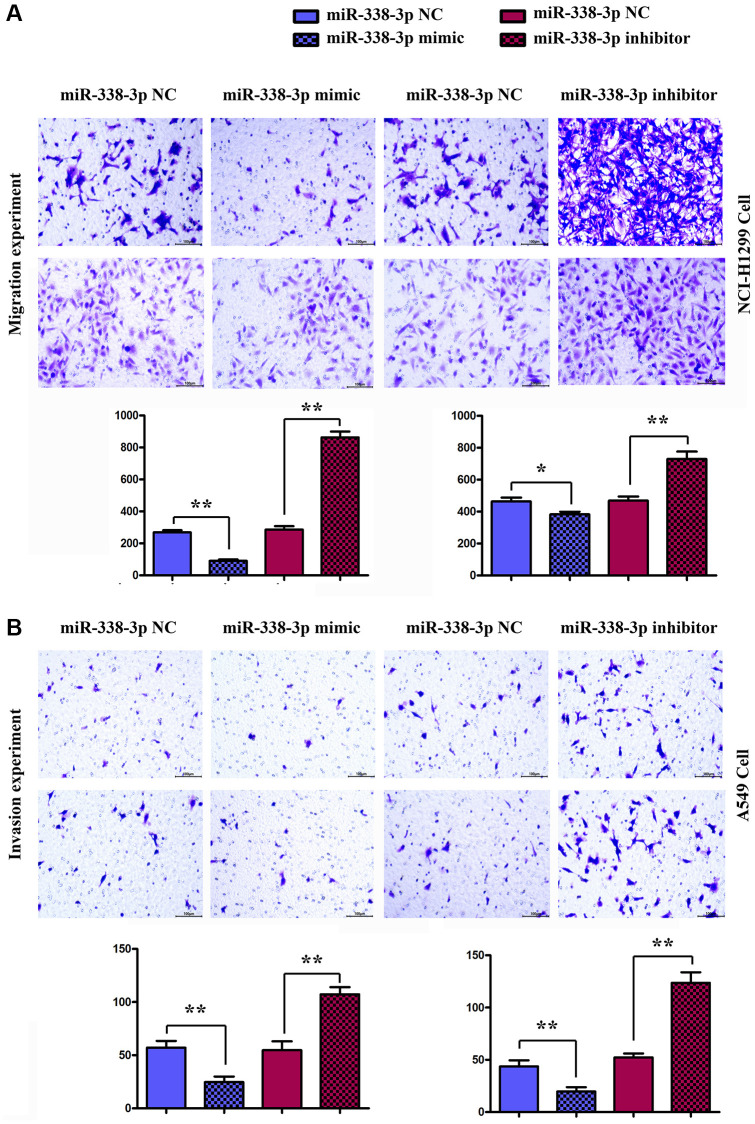
**Detection of the number of migrating and invasive NCI-H1299 and A549 cells in each group by migration and invasion experiments.** (**A**) Migration experiment shows the number of migrating NCI-H1299 and A549 cells (×400). ^**^P < 0.01 means that the difference is statistically significant. (**B**) Invasion experiment reveals the number of invasive NCI-H1299 and A549 cells (×400). ^**^P < 0.01 means that the difference is statistically significant.

**Figure 7:**
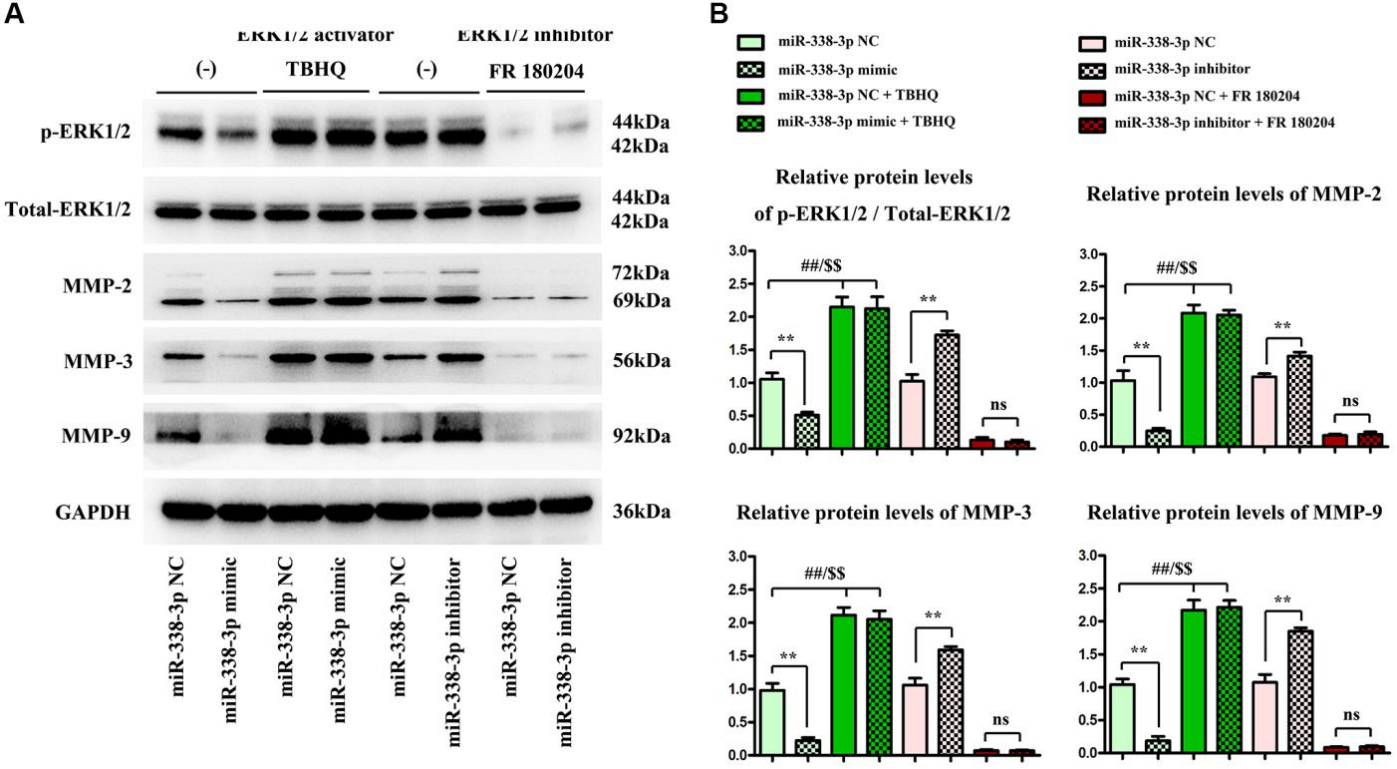
**Overexpressed miR-338-3p affects the expression of proteins related to migration and invasion function of NCI-H1299 and A549 cells through the ERK pathway.** (**A**) Protein expression levels of p-ERK1/2, t-ERK1/2, MMP-2, MMP-3 and MMP-9 in NCI-H1299 and A549 cells. (**B**) Statistical analysis results of protein expressions in NCI-H1299 and A549 cells. ^**^P < 0.01, ^##^*P* < 0.01 and ^$$^*P* < 0.01 mean that the difference is statistically significant.

Migration assay (Figure 5A) and invasion assay (Figure 5B) illustrated that the number of cells passing through the lower chamber and basement membrane in miR-mimic group was markedly smaller than that in NC mimic group, and the number of cells passing through the lower chamber and basement membrane in miR-inhibitor group was remarkably larger than that in NC inhibitor group. Western blotting assay (Figure 6A, 6B) confirmed that after exogenous overexpression/mimic of miR-338-3p, the protein expressions of MMP-2, MMP-3 and MMP-9 in LUAD A549 cells notably declined. Additionally, compared with that in NC inhibitor group, the expression levels of proteins related to the migration and invasion function of A549 cells in miR-inhibitor group were significantly increased. These results indicated that the overexpression of miR-338-3p can inhibit the migration and invasion of LUCA cells (Figures 6 and 7).

### Overexpression of miR-338-3p inhibited the invasion and migration of LUCA cells by regulating ERK1/2 phosphorylation

Transwell migration and invasion experiments showed that the number of cells crossing the lower chamber and basement membrane in miR-mimic group was markedly smaller lower than that in NC mimic group (*P* < 0.05), the number of cells crossing the lower chamber and basement membrane in miR-inhibitor group was dramatically increased compared with that in NC inhibitor group. It can be concluded that the overexpression of miR-338-3p can inhibit the migration and invasion of LUAD cells.

Meanwhile, ERK1/2 agonist TBHQ and ERK1/2 inhibitor FR180204 [12] were added to further verify that miR-338-3p affected the expression of proteins related to the migration and invasion function of A549 cancer cells through the ERK pathway. As shown in Western blotting assay, the protein expressions of MMP-2, MMP-3 and MMP-9 in LUAD cells significantly declined after exogenous overexpression of miR-338-3p. Compared with those in NC inhibitor group, the expression levels of proteins related to migration and invasion function of A549 cells in miR-inhibitor group were obviously increased. After the addition of TBHQ, the protein expressions of p-ERK1/2, t-ERK1/2, MMP-2, MMP-3 and MMP-9 in miR-mimic group had no significant changes compared with those in NC mimic group. After the addition of FR180204, there were no significant changes in the expressions of these proteins in miR-inhibitor group compared with those in NC inhibitor group. These findings suggested that miR-338-3p affects the migration and invasion of LUAD cells through the ERK pathway (Figure 7A, 7B).

### Overexpression of miR-338-3p inhibited the proliferation of LUAD cells by suppressing cyclin A2 and cyclin D1

**Figure 8:**
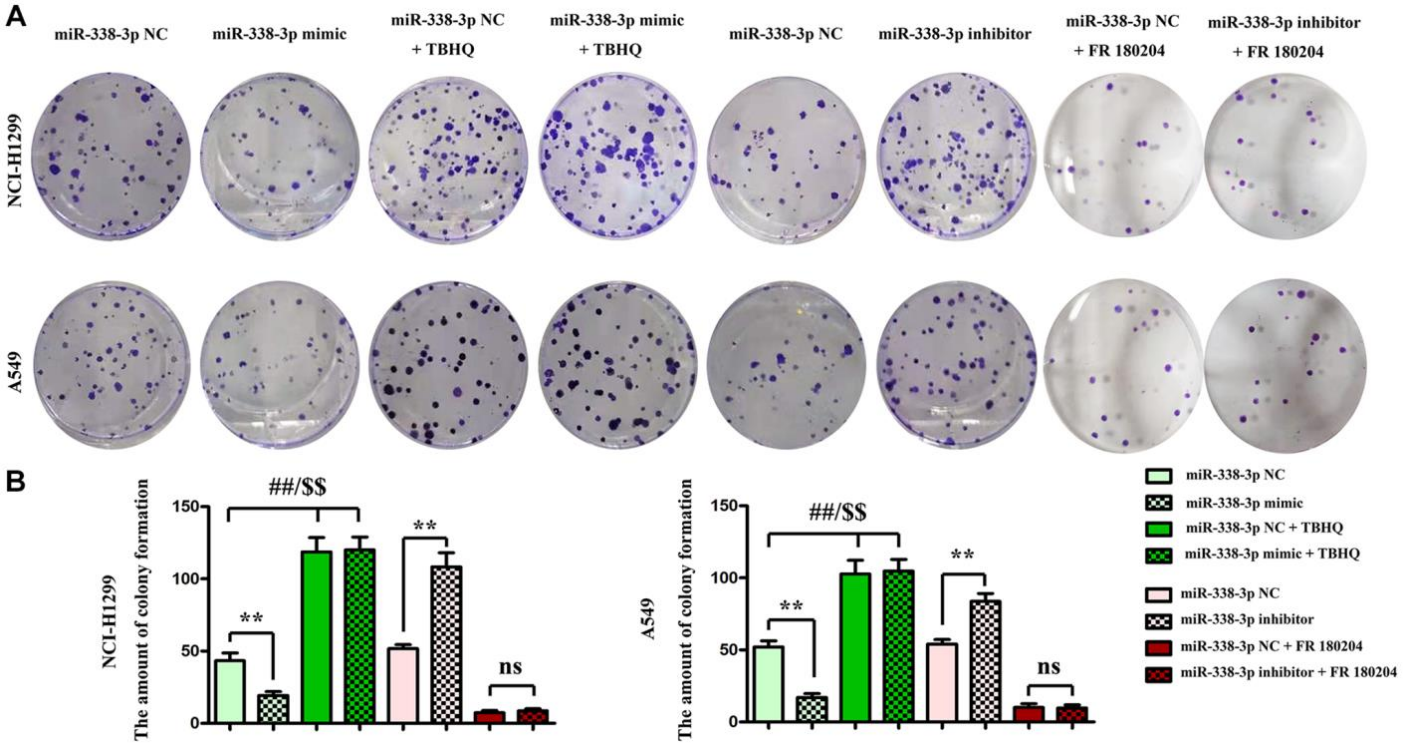
**Regulatory roles of miR-338-3p on the colony formation ability of NCI-H1299 and A549 cells dependent on the ERK signal.** (**A**, **B**) The number of colonies formed after transfection with miR-338-3p mimic and inhibitor treated with FR180204 or TBHQ. ^**^P < 0.01, ^##^*P* < 0.01 and ^$$^*P* < 0.01 mean that the difference is statistically significant.

**Figure 9:**
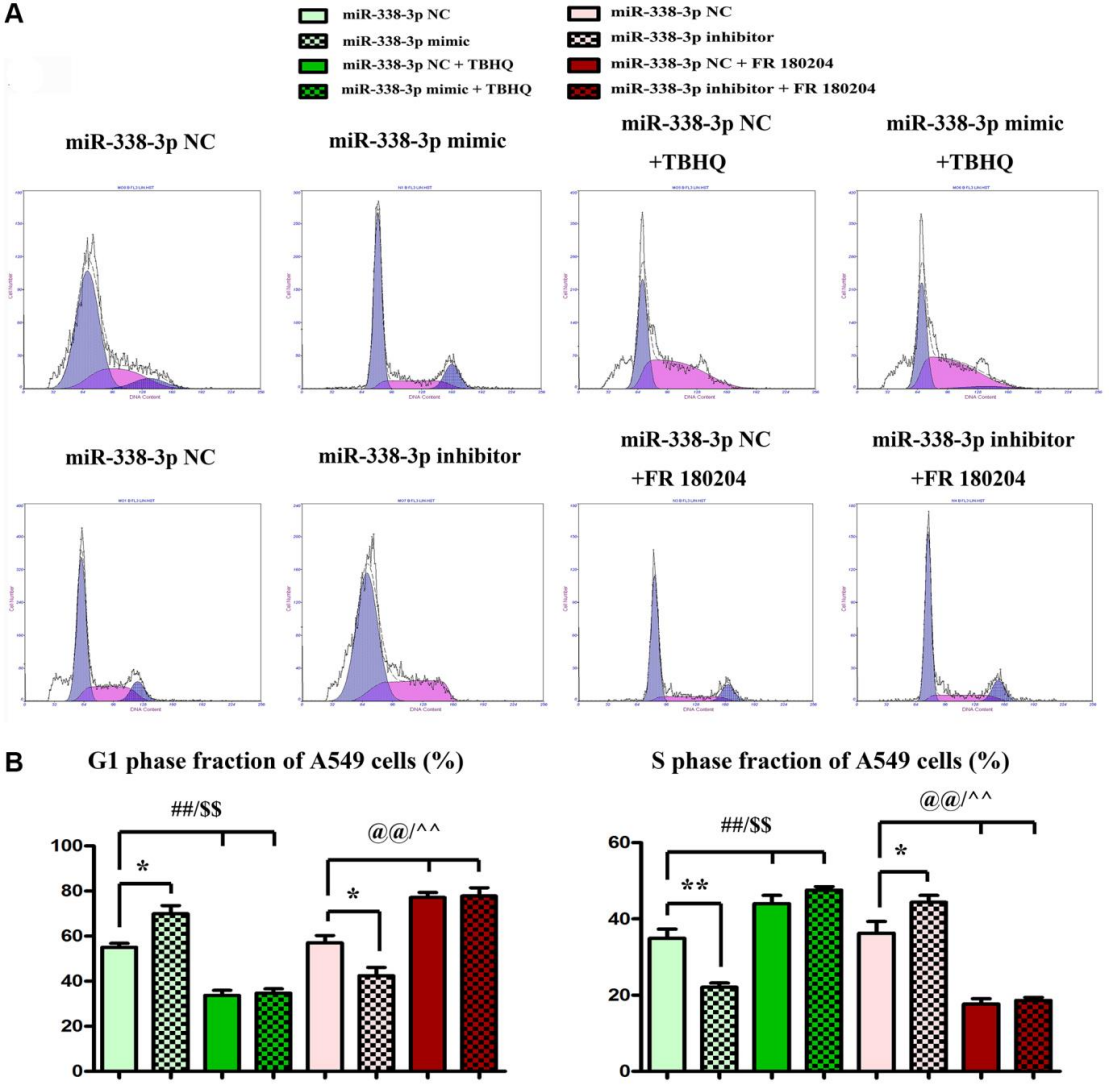
**Overexpression or suppression of miR-338-3p inhibits or increases the proliferation of LUAD A549 cells by regulating ERK1/2 phosphorylation.** (**A**, **B**) ^**^P < 0.01, ^##^*P* < 0.01 and ^$$^*P* < 0.01 mean that the difference is statistically significant.

**Figure 10:**
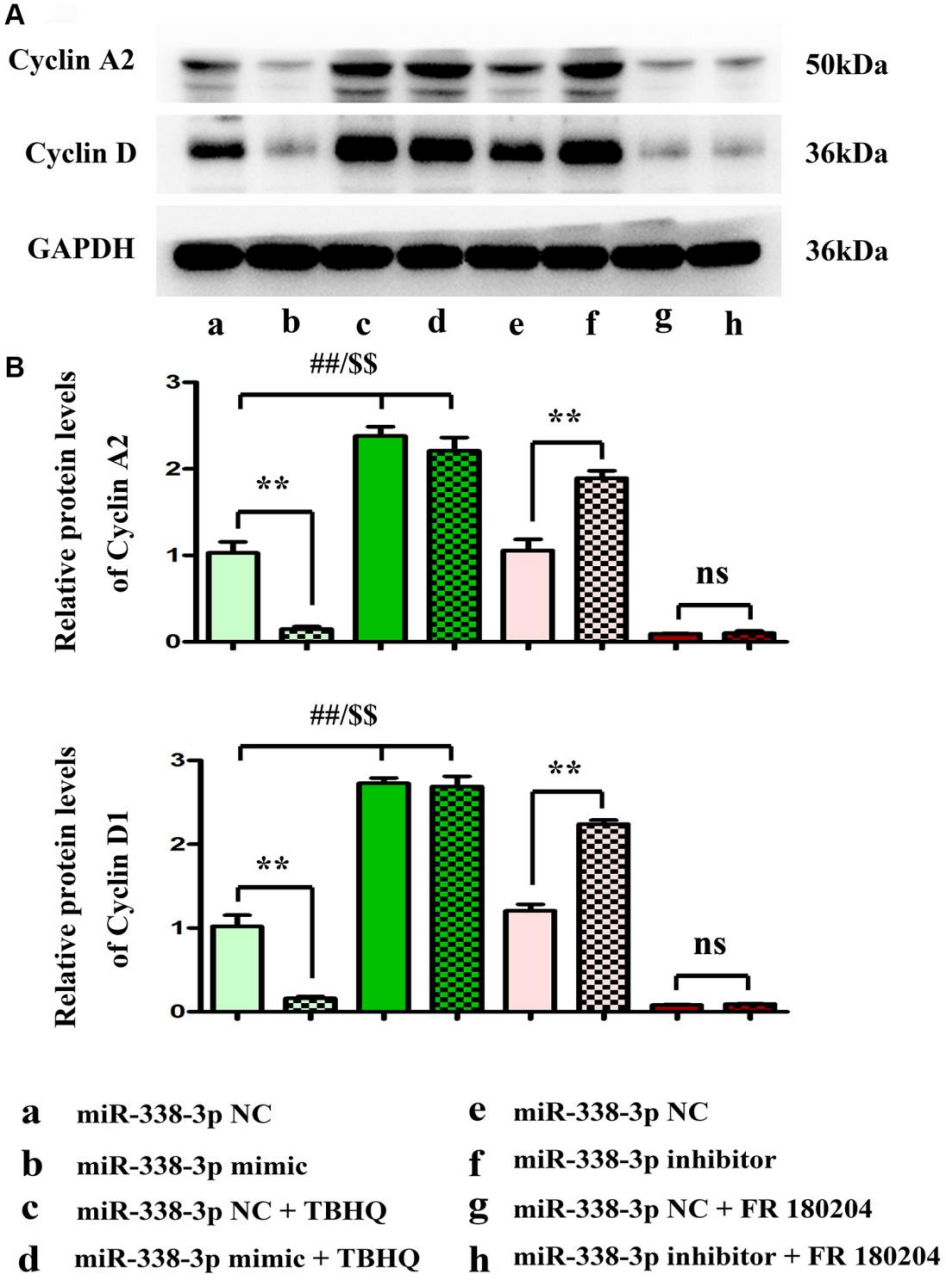
**miR-338-3p modulates the proliferation of A549 cells by regulating the expression of cyclin A2 and cyclin D1, and these results are dependent on the ERK1/2 phosphorylation.** (**A**, **B**) ^**^P < 0.01, ^##^*P* < 0.01 and ^$$^*P* < 0.01 mean that the difference is statistically significant.

The proliferation ability of LUAD cells was detected by colony formation assay. Compared with NC mimic group, the number of colonies formed by NCI-H1299 cells as well as A549 cells which were cloned in miR-mimic group declined remarkably, suggesting that miR-338-3p overexpression can inhibit the proliferation of tumor cells. However, the miR-inhibitor group had more colonies formed by LUAD cells which were cloned than NC inhibitor group, these results were shown in Figure 8A, 8B. At the same time, the cell cycle was analyzed by flow cytometry. Compared with NC groups, the percentage of cells arrested in G1 phage was increased, while the ratio of cells in S phage was decreased in the miR-mimic group, but they were contrary in miR-inhibitor group, and their roles were reversed by ERK1/2 activator or inhibitor (Figure 9A, 9B). Furthermore, Western blotting assay was conducted to detect the expression of cell proliferation-related proteins, and the results suggested that compared with those in NC mimic group, the protein expression levels of cyclin A2 and cyclin D1 in A549 cells in miR-mimic group were dramatically reduced, but they were raised remarkably after A549 cells were transfected with miR-inhibitor. The effects of miR-338-3p were rectified by ERK1/2 activator TBHQ, and the roles of miR-338-3p inhibitor were reversed by ERK1/2 inhibitor FR180204 (Figure 10A, 10B). These results indicated that the modulatory roles of miR-338-3p were dependent on the activation of ERK1/2.

## DISCUSSION

Lung cancer is a common malignant tumor of respiratory system. According to statistics, the incidence of lung cancer is the highest in male malignant tumor patients, second to female patients, and its mortality is the highest in both male and female malignant tumor patients. Tumor invasion and metastasis are frequently seen at the initial diagnosis of lung cancer and serve as leading causes of lung cancer-related death [11]. The regulatory effect of miRNAs in lung cancer has been widely reported. For example, miR-193 blocks invasion and migration of NSCLC [12]. As a prognostic factor, miR-454 contributes to the proliferation and metastasis of lung cancer cells [13]. With regard to human cancer studies, abnormal down-regulation of miR-338-3p has been demonstrated in a variety of human malignancies, such as ovarian, colorectal and cervical cancers [14], and miR-338-3p acts on the SOX5/Wnt/beta-catenin, ADAM17 and ZEB2 proteins to suppress the progression of various cancers. Moreover, these target proteins are directly or indirectly associated with MAP3K2-ERK1/2 signaling pathway [15–21].

MiRNAs inhibit target gene expression at the post-transcriptional level by partially complementary pairing with target gene mRNAs. Studies have shown that miRNAs are involved in various biological processes, including cell proliferation, apoptosis, differentiation, metabolism and development and tumor metastasis. However, miRNAs are not completely matched with their target genes, so that it is difficult to determine miRNA target genes. Through the analysis on the known miRNAs and their target genes, researchers found that the 3′-UTR of the target genes has a complete pairing region (2–8 nt) with at least 7 consecutive nucleotides at the 5′ end of the miRNAs. This part of miRNA sequences is called the “seed sequence”. The complementary region between the mRNA and the miRNA seed sequence is often conservative in species.

The results of this study showed the low expression of miR-338-3p in LUAD. Transwell migration and invasion experiments denoted that overexpression of miR-338-3p can inhibit the migration and invasion of LUAD cells. MiR-338-3p directly combines with 3′-UTR of MAP3K2 and results in the degradation of MAP3K2. Moreover, the activation of p-ERK1/2 is under the control of MAP3K2 via its regulatory roles for NF1, K-Ras and PP2A [22, 23] MAP3K2-ERK1/2 signaling pathway is thought to modulate the activation or expression of the STAT3, MMPs, survivin and cyclin D1, thus facilitating the invasion, metastasis and proliferation of cancer cells. Therefore, the relationship between miR-338-3p and MAP3K2/ERK may powerfully influence the tumor deterioration. [22–24] The results of this study elucidated that the overexpression of miR-338-3p may inhibit the phosphorylation of the ERK1/2 signaling pathway by regulating the protein expression of MAP3K2 and improve the invasion and metastasis of LUAD cells.

In the present study, the results revealed that miR-338-3p significantly reduced the expressions of these proteins, and inhibited the migration and invasion of NCI-H1299 and A549 cells. MMPs are zinc-containing enzymes that degrade all extracellular matrix (ECM) components, which are crucial for the invasion and metastasis of most tumor cells [25]. Normally, MMPs are involved in wound repair, tissue regeneration and reproduction [26] as well as carcinogenesis, including the growth, differentiation, migration, apoptosis, invasion and metastasis of cancer cells [27]. The structure of MMPs in the catalytic area contains cysteine insert, which is generally manifested as the peptide structure under physiological resting state. After the MMPs are activated, the insert can be combined with gelatin, collagen and elastin, crack the degraded ECM, adjust tumor cell adhesion, and promote angiogenesis and cell invasion [28]. MMP-2 and MMP-9, also known as gelatinases, have been long recognized as major contributors to the proteolytic degradation of ECM during tumor invasion, which are responsible for mediating the degradation of different components of the ECM, the shackle for tumor [29]. MMPs can activate the TGF-β/Smad signaling, which is a powerful modulatory signal to enhance tumor invasion, and can also affect tumor cell behaviors and lead to cancer progression [30, 31]. Considering that ERK1/2 signal is a pivotal and powerful regulatory factor for the expression of MMPs [32–34], it is speculated that the inhibitory effect of miR-338-3p on LUAD invasion may be dependent on ERK1/2 activation. Meanwhile, the addition of ERK1/2 agonist TBHQ and ERK1/2 inhibitor FR180204 further verified that miR-338-3p affected the expression of MMP proteins related to the migration and invasion function of cancer cells through the ERK pathway. These results suggested that miR-338-3p affects the protein expressions of MMP-2, MMP-3 and MMP-9 in LUAD cells through the ERK pathway.

After transfection with miR-338-3p mimic, the protein expressions of cyclin A2 and cyclin D1 markedly declined, and the overexpression of miR-338-3p suppressed the proliferation of NCI-H1299 and A549 cells. Among them, cyclin A2 is the first cyclin [35] that has been found to be associated with malignant transformation of cells [36]. Currently, it has been confirmed that cyclin A2 is highly expressed in multiple tumor tissues, while it is not expressed or low expressed in normal tissues [36, 37]. The results of this study displayed that cyclin A2 was also highly expressed in LUAD, and the overexpression of miR-338-3p could inhibit its protein expression. Cyclin D1 as an important gene in cell cycle mainly acts on G1 phase and is viewed as a crucial player in regulating cell cycle. Highly expressed cyclin D1 can promote cell proliferation and differentiation and is closely related to the occurrence and development of tumors [38]. In addition, cyclin D1 plays a significant role in the stability of chromosomes and is an important gene leading to chromosome instability and malignant transformation. Studies have discovered that cyclin D1 level is closely correlated with tumor stage, degree of differentiation and lymphatic metastasis, and it is considered to be an important indicator for evaluation of tumor malignancy [39]. In this study, the protein levels of cyclin A2 and cyclin D1 in LUAD cells were markedly increased after the exogenously low expression of miR-338-3p. Cyclin D1 and cyclin A2 are also controlled by MAP3K2-ERK1/2 signal [40–42] Therefore, after TBHQ was added, the protein expressions of cyclinA2 and cyclin D1 in miR-mimic group did not change significantly compared with those in NC mimic group, and FR180204 reversed these results. Based on these, it can be concluded that miR-338-3p affects the proliferation of NCI-H1299 and A549 cells through the ERK pathway.

In conclusion, miR-338-3p can regulate the ERK1/2 signaling pathway by targeting MAP3K2, and inhibit the expressions of downstream cell migration, invasion and proliferation-related proteins, thus playing an important role in the proliferation, invasion and metastasis of LUAD cells. Therefore, miR-338-3p may be one of the biological markers for tumor diagnosis and prognosis evaluation. However, whether miR-338-3p can serve as a target for early screening and targeted therapy of tumors needs further research.
